# Effect of unilaterally manipulating the step length of an embodied avatar on gait symmetry in healthy adults

**DOI:** 10.1007/s10055-026-01406-2

**Published:** 2026-06-25

**Authors:** Iris Willaert, Rachid Aissaoui, Valentin Vallageas, Sylvie Nadeau, Cyril Duclos, David R. Labbe

**Affiliations:** 1 Open Innovation Laboratory in Health Technologies (LIO), École de technologie supérieure, Montréal, Canada; 2 Laboratoire de Pathokinésiologie de Montréal, Institut Universitaire sur la Réadaptation en Déficience Physique de Montréal, Université de Montréal, Montréal, Canada; 3 CHUM Research Center, Université de Montréal, Montréal, Canada

**Keywords:** Virtual reality, Embodiment, Gait symmetry, Self-avatars, Gait rehabilitation

## Abstract

In virtual reality, mapping one’s body movements to an avatar creates a sense of embodiment, where the avatar is perceived as part of one’s own body. Previous studies have indicated that introducing a spatial offset between the avatar’s movements and the user’s real movements can lead to users unconsciously adjusting their movements to align with the avatar. However, while the effects of such spatial distortion have been studied in the movement of the upper body, little is known about its influence on gait. This study aimed to assess whether altering the step length of an embodied avatar induces a more asymmetrical walking pattern. Thirty healthy participants underwent a treadmill walking experiment while wearing a head-mounted display that displayed an embodied avatar mirroring their movements from a first-person perspective. An algorithm was developed to dynamically increase one of the avatar’s step lengths in real-time, introducing gait asymmetry. The effect of step length manipulation on gait asymmetry was assessed under two conditions, where the avatar’s step length was gradually increased unilaterally for either the dominant or non-dominant leg. Results indicated that participants did not significantly adjust their gait symmetry to match the avatar’s increased step length, whether it was applied to the dominant or non-dominant leg. These findings suggest that healthy participants may be more resistant to visual feedback-induced changes provided by an embodied avatar, in contrast to the adjustments observed in upper-body movements.

**Supplementary Information:**

The online version contains supplementary material available at https://doi.org/10.1007/s10055-026-01406-2.

## Introduction

Virtual reality (VR) has emerged as a therapeutic tool for the assessment and treatment of patients with neurological conditions who suffer from walking impairments (Porras et al. 2019; Zhang et al. 2021; Keshner and Lamontagne 2021). Patients with neurological disorders, such as those resulting from a stroke, often face gait deficits that significantly affect their quality of life (Arene and Hidler 2009; Mayo et al. 1999). Rehabilitation programs typically utilize motor learning principles, focusing on intense, task-oriented, and repetitive training tailored to each patient’s specific needs (Belda-Lois et al. 1999). Providing visual feedback during gait has shown to be such an effective interactive method to engage patients during rehabilitation and promote the process of motor learning (Kim 2012; Maestas et al. 2018; Ramachandran and Altschuler 2009). Research has shown that augmenting visual feedback can influence gait symmetry, even in healthy individuals. For instance, Kim and Krebs (Kim 2012) presented visual feedback on a screen to participants as two separate vertical bars corresponding to the left and right step lengths during treadmill walking in healthy participants. When this feedback was distorted by lengthening one of the bars to create a perceived asymmetry in step length, participants spontaneously adjusted their gait away from symmetry. This adjustment likely occurred due to the fact that the distortion of the bars creates a prediction error between the actual step length of participants and where the bar is displayed on the screen, as the latter is not where it is supposed to be. In response, participants adjusted their step lengths to align them with the displayed feedback (Kim 2012).

Immersive VR environments offer the added possibility to create visual illusions about the body that “bend the truth,” delivering real-time altered visual feedback of movements which is not possible in the real world (Porras et al. 2018; Adamovich et al. 2009). Furthermore, virtual bodies or self-avatars can represent the user’s body movements inside the virtual environment seen from a first-person perspective position (Slater et al. 2010). As this happens in real-time, the user is in control of the avatar’s movements, which can lead to the acceptance of the virtual body as part of their own body and typically creates a sense of embodiment (SoE) over the avatar (Kilteni and Slater 2012; Kokkinara and Slater 2014). Extensive research has demonstrated that SoE can be a particularly strong illusion and plays an important role in users’ experience within immersive VR (Slater et al. 2008; Banakou et al. 2013; Kokkinara et al. 2016; Peck et al. 2013). More recently, evidence showed that manipulating alignment by introducing a spatial offset between the avatar and the user can affect the user’s motor behaviour without breaking the sense of embodiment. This phenomenon, referred to as the *self-avatar follower effect* by Gonzalez-Franco et al. (Gonzalez-Franco et al. 2020), describes how users unconsciously adjust their movements to align with the avatar’s altered position. This has been demonstrated in upper body reaching tasks, where participants adapted their movements to mirror the virtual body’s misalignment. Moreover, this adjustment occurred spontaneously, without explicit instructions, while maintaining a strong sense of embodiment over the avatar (Burin et al. 2019; Bourdin et al. 2019; Gonzalez-Franco et al. 2020; Cohn et al. 2020).

These previous studies have shown that users tend to follow their avatars and unconsciously adapt their movements in response to altered avatar movements. However, these effects have mostly been observed during discrete, visually guided upper-limb tasks, such as reaching, where movements are directed toward a visible target. (Burin et al. 2019), in contrast, demonstrated that this kind of motor interference can also occur during continuous, rhythmic motion. In their study, participants asked to draw straight lines while observing an avatar performing elliptical movements gradually adjusted their trajectories to match the avatar’s motion. Importantly, this occurred even in the absence of an explicit target and was stronger when participants reported a higher sense of ownership over the avatar.

To our knowledge, no previous work has explored whether this “follower effect” also applies to gait, a similarly rhythmic and continuous form of movement, but one that typically lacks a visual goal and is governed by different motor control mechanisms. Gait is driven by lower-level control processes, including central pattern generators, and involves the lower limbs (Malone and Bastian 2010; Clark 2015), which have been less studied in this context. While the findings from Burin et al. (2019) suggest that the follower effect could extend to gait, it remains an open question whether participants will spontaneously adapt their walking patterns to follow distorted avatar movements during locomotion.

This study directly addresses that question. It builds upon our previous work using the same experimental dataset (Willaert et al. 2024), but with a different focus and a set of hypotheses defined during the original study design (documented prior to data collection), although the study was not preregistered. Whereas the first study examined how gait distortions were perceived by participants and how embodiment changed as a function of distortion amplitude, particularly whether embodiment was maintained beyond the point of detection, this paper investigates whether the follower effect, as described by Burin et al. (2019) and others, can be observed in the context of gait. Specifically, we examine whether participants follow changes in the avatar’s step length, and how the level of embodiment may influence the extent to which they adapt their own gait in response.

A virtual environment was developed in which participants walked on a treadmill while embodied in a full-body avatar whose step length was altered. The step length distortion was applied unilaterally,and was deliberately chosen to introduce a controlled left–right asymmetry in the avatar’s gait, allowing us to test whether an asymmetric spatial distortion applied to a single limb is sufficient to elicit a self-avatar follower effect during gait. We applied the distortion to each leg separately (dominant and non-dominant) to examine whether responses depend on laterality.

Both the dominant and non-dominant legs were studied as prior research has highlighted that limb differences exist during able-bodied walking, potentially influenced by limb dominance (Sadeghi et al. 2000). Additionally, proprioceptive acuity has been shown to differ between the dominant and non-dominant lower limbs (Han et al. 2013), suggesting that each leg may respond differently to step length distortions.

It was hypothesized that the gradual manipulation of the avatar’s step length would prompt participants to follow the distorted avatar’s step length and adjust their own step length, thereby inducing asymmetrical gait patterns (H1). Furthermore, in line with previous findings that detection of step length distortions did not affect embodiment (Willaert et al. 2024), we hypothesized that changes in gait symmetry remain past the detection threshold (H2).

## Related work

### Embodiment

The sense of embodiment has been defined as the perceptual feeling of having a virtual body, arising from three main components: body ownership, agency, and self-location (Kilteni and Slater 2012). The sense of body ownership refers to the feeling that the virtual body is one’s own. The sense of agency refers to the feeling of controlling the virtual body’s movements, i.e., that one can initiate and regulate its actions. Finally, self-location refers to the feeling of being spatially located within the virtual body. Numerous studies have shown that the illusion can be induced towards avatars of various bodily sizes (Banakou et al. 2013; Abtahi et al. 2019) body shapes (Kilteni et al. 2012; Normand et al. 2011), different genders (Bolt et al. 2021) or ethnicities (Peck et al. 2013). Even avatars with physically impossible features, such as a third (Drogemuller et al. 2019), an unusually long arm (Kilteni et al. 2012) or an enlarged lower limb (Vallageas et al. 2024) can be incorporated into an individual’s body representation. This ability of VR to reshape body representation is further illustrated through phenomena like the proprioceptive drift. Proprioceptive drift refers to the shift in an individual’s perceived location of their own body parts toward the position of the virtual body or avatar, driven by the integration of multisensory cues (Botvinick and Cohen 1998). For instance, in studies involving the rubber hand illusion (Botvinick and Cohen 1998), participants reported a shift in the perceived position of their real hand toward a fake hand when synchronous visuotactile stimuli were provided (Tsakiris and Haggard 2005). Moreover, this ownership illusion is not limited to proprioceptive drift but extends to physical drift, where participants unconsciously physically move their real hands toward the rubber hand (Asai 2014).

Proprioceptive drift has also been observed during embodiment of a virtual avatar, where users recalibrate their body representation during static postures and active movements. When interacting with avatars in VR, participants often perceive their real limbs as being closer to the avatar’s position (Kilteni et al. 2012; Sanchez-Vives et al. 2010; Brugada-Ramentol et al. 2019). During active movements, proprioceptive drift has been demonstrated where participants’ perceived limb position shifts toward the position of a virtual avatar as they perform goal-directed tasks, such as reaching or manipulating objects (Shibuya et al. 2018; Kalckert and Ehrsson 2012). Notably, this effect has been found to be more pronounced in the moving rubber hand illusion compared to the classic static version (Riemer et al. 2013). This drift has also been demonstrated in other parts of the body, including the foot, through the Rubber Foot Illusion. In this paradigm, participants perceive their real foot as shifting toward the location of a rubber foot when synchronous visuotactile stimulation is applied (Crea et al. 2015; Matsumoto et al. 2020) with similar illusion strength as in the rubber hand (Flögel et al. 2016). Moreover, synchronous movement between the real and rubber foot enhances the sense of ownership and results in a more pronounced proprioceptive drift compared to static conditions (Teaford et al. 2021).

When there is a spatial incongruency between the virtual body and the real body position, experienced from a first-person perspective, a sense of ownership can persist as long as synchronous sensory cues are maintained (Maselli and Slater 2014).For example, a 25 cm lateral offset reduced ownership without eliminating it, whereas larger misalignments (e.g., 80 cm) significantly weakened the illusion, as the virtual body no longer aligned with the proprioceptive estimate of position of their body position. The sense of self-location proved more resilient to such discrepancies, with participants reporting a feeling of being located near the virtual body even when ownership diminished.

Together, these findings show that embodiment can extend to the lower limb and can recalibrate perceived limb position through multisensory integration (e.g., proprioceptive drift). Importantly, embodiment can also persist despite moderate spatial mismatches between the virtual and real body, as long as synchronous sensory cues are maintained. This resilience is central to the present work: it suggests that we can introduce controlled distortions to an embodied avatar while still preserving a sense of ownership and self-location, creating the conditions under which altered avatar feedback may influence motor behavior.

### Altered avatar feedback and movement adaptation

In virtual environments where a tracked avatar mirrors the user’s movements in real time, users typically experience a strong sense of embodiment and agency (Spanlang et al. 2014). Under these conditions, the avatar’s displayed motion is not merely perceptual: recent work shows it can also act as a cue that shapes motor behaviour.

This has been observed when the avatar’s movements are deliberately altered to create a deviation from what is actually being produced by the users (Gonzalez-Franco et al. 2020; Bourdin et al. 2019; Burin et al. 2019; Berger et al. 2022). For instance, in a study where participants were tasked with drawing straight lines while observing an avatar executing elliptical movements, they tended to unconsciously adjust their movements to resemble the avatar’s actions, resulting in drawing shapes more similar to the avatar’s ellipses (Burin et al. 2019). This mismatch, where the virtual hand’s observed position differed from the real hand’s sensory feedback, demonstrated how the avatar’s movements influenced motor behaviour, prompting participants to adjust their own movements to align more closely with the avatar. Moreover, these effects were significantly higher when the avatar was perceived as part of their own body, demonstrating these incongruent errors are perceived differently depending on the level of embodiment. Prediction errors become more relevant in the context of sensorimotor reweighting when the avatar’s body is strongly perceived as the user’s own body (Burin et al. 2019).

A similar phenomenon was observed during a seated arm-reaching task, where participants reached for a visual target while the avatar’s hand was snapped to a predefined trajectory, introducing a spatial mismatch of a maximum of 30 degrees in the medio-lateral axis between their real and the avatar’s arm movements. Despite not being explicitly instructed to do so, participants unknowingly adjusted their arm movements to align more closely with the avatar’s altered position, rather than continuing to reach in a straight line, all while maintaining their sense of embodiment (Gonzalez-Franco et al. 2020). This effect was later extended to vertical offsets, where participants adapted to spatial displacements of up to $15^\circ$ along the vertical axis (Cohn et al. 2020). The authors further suggested that observed motor adjustments are a by-product of embodiment. Their findings showed that participants reported higher embodiment when the spatial offset was introduced gradually rather than instantaneously. Greater embodiment was associated with stronger motor adjustments, as participants instinctively aligned their movements with the avatar to minimize the spatial mismatch. In contrast, when the distortion was introduced instantaneously, the resulting sensory conflict reduced the sense of embodiment and reduced the observed motor adjustments, although embodiment was not entirely disrupted due to the preserved visuomotor synchronization between real and avatar movements.

This adaptation phenomenon was also demonstrated in the "Pinocchio Illusion," where participants’ real arm movements extended further along the anterior-posterior axis to align with the exaggerated elongation of the virtual arm and nose (Berger et al. 2022). In this study, participants engaged in a task where they repeatedly tapped the virtual avatar’s nose. With each tap, the virtual arm elongated by up to 50% of its original length, and the virtual nose gradually extended forward to a maximum of 70 cm, creating the illusion of artificial lengthening. Moreover, this effect was strongly influenced by the synchrony of feedback: when visual and proprioceptive feedback were synchronized in real-time with participants’ tapping movements, a stronger sense of ownership over the virtual arm, which was accompanied by more pronounced drift in their real arm movements. In contrast, asynchronous feedback, introduced by delaying the visual elongation relative to the participant’s tapping, diminished the illusion of embodiment and reduced the observed motor adjustments.

High embodiment scores for ownership and agency were also observed during an elbow flexion task where the avatar’s arm movement amplitudes were modulated. In this study, participants were instructed to bring their elbow to a $90^\circ$ flexion while the avatar’s arm flexion was altered without their explicit knowledge (Bourdin et al. 2019). When the participant’s real arm reached the $90^\circ$ position, the avatar’s arm was either modulated to $75^\circ$ ($-15^\circ$) or $105^\circ$ ($+15^\circ$) with respect to the real arm. Results indicated that when the avatar’s arm showed a reduced range of motion ($75^\circ$), participants compensated by increasing their actual elbow flexion. Conversely, when the avatar’s arm displayed an over-extended range of motion ($105^\circ$), participants responded by decreasing their real elbow flexion. These findings suggest that altered visual feedback, when experienced with a strong sense of ownership, can significantly influence motor performance.

The motor compensation observed in the above-mentioned studies (Burin et al. 2019; Bourdin et al. 2019; Berger et al. 2022; Gonzalez-Franco et al. 2020; Cohn et al. 2020) may be explained by the theories of motor control, particularly those involving the forward model inherent in voluntary motor control (Welniarz et al. 2021; Miall and Wolpert 1996). The introduced spatial offset creates a disparity between the predicted limb position (actual limb positions) and the visual information provided by the avatar’s position. This mismatch likely triggers a sensory conflict, compelling the nervous system to minimize the error by adjusting limb movements to align more closely with the visual feedback (Maselli et al. 2022). Interestingly, in these studies (Burin et al. 2019; Bourdin et al. 2019; Berger et al. 2022; Gonzalez-Franco et al. 2020; Cohn et al. 2020), participants were generally unaware of their adjusted motor performance but consistently reported high levels of embodiment. As suggested by the authors, when actions produce unexpected results, as observed in these tasks (such as an altered embodied avatar), the brain updates its internal models to reduce prediction errors while preserving a coherent sense of the body. These adjustments are driven by the need to resolve sensory discrepancies rather than intentional action, which may explain why they are not consciously perceived (Maselli et al. 2022). Moreover, (Burin et al. 2019) demonstrated that these motor adjustments can occur even when participants are explicitly aware of the experimental manipulations. This finding may imply that conscious awareness may be more closely tied to intended actions than to actually executed movements.

Moreover, the motor adjustments observed in VR embodiment settings when exposed to spatially offset avatars may offer possible implications for motor rehabilitation. By providing real-time, distorted visual feedback, therapists can present patients with customized manipulations of their avatar’s movements tailored to specific therapeutic goals (increasing range of motion or improving gait symmetry). This approach can be utilized to intentionally amplify errors made during motor tasks, highlighting movement discrepancies to enhance awareness and promote cognitive engagement in correcting those errors (Gaveau et al. 2014; Fasola et al. 2019). Alternatively, the avatar’s movements can be programmed to align more closely with the intended motion, guiding patients toward improved motor performance. In the context of gait rehabilitation, where abnormalities such as step length asymmetry are commonly observed (Balasubramanian et al. 2007; Olney and Richards 1996), an avatar’s step length can be strategically exaggerated or reduced in real time to encourage patients to adjust their own step length in response, promoting gait symmetry. However, to effectively apply such interventions, it is crucial to first understand how healthy individuals respond to step length distortions and whether they adjust their gait symmetry.

The effects of spatially offset avatars on motor adjustments have primarily been studied in upper-body movements, particularly in the context of reaching tasks. Although prior studies have demonstrated that lower-limb motor behavior can be influenced by self-avatar movements, these effects were primarily observed in the context of temporal mismatches or decision uncertainty, for example, ambiguity about when to initiate a step or which movement to perform when synchronizing with a delayed avatar or reproducing memorized movement sequences, rather than spatial distortions (Boban et al. 2024, 2023). For instance, Boban and colleagues showed that participants unintentionally synchronized their stepping with a temporally delayed avatar and were influenced by avatar errors during memorized movement sequences. However, research investigating motor adaptation to spatially distorted lower-limb avatar movements, especially during gait, remains limited. This gap in the literature highlights the need to examine whether the mechanisms of adaptation observed in upper-limb tasks also occur during gait in response to spatially distorted avatar movements.

Taken together, prior work on the self-avatar follower effect suggests that gradually introduced distortions under strong embodiment can elicit spontaneous motor adjustments. In the present study, we test this prediction in gait by applying unilateral step-length distortions to an embodied avatar during treadmill walking. Unlike visually guided upper-limb reaching, gait is a rhythmic lower-limb task that typically lacks an explicit visual target and is governed by partly distinct motor control mechanisms (Hajnal et al. 2007). Therefore, it remains unclear whether similar avatar-based motor adjustments would occur during locomotion.

## Methodology

### Participants

Thirty participants with no lower limb injuries that affected their gait volunteered to take part in this study. The target sample size (N = 30) was established during study planning based on comparable treadmill studies examining altered visual feedback and step-length asymmetry in healthy adults (Kim 2012; Tobar et al. 2018; Sato et al. 2022). Given the within-subject repeated-measures design, this sample size was considered appropriate for detecting within-participant changes in gait symmetry.Data from one participant was excluded due to technical issues during the experiment. The analysis focused on the remaining 29 participants, consisting of 9 females and 20 males, with an average age of 26.1 years (± 4.1), height of 174.6 cm (± 10.2), and weight of 71.6 kg (± 12.2). The participants had an average self-selected gait speed of 1.20 m/s (± 0.1). Lower limb dominance was established by instructing participants to kick a foam ball toward the wall, and the foot they naturally used for the kick was considered their dominant one. Three participants were left-footed. Ethical approval for the study was obtained from the ethics committees of CHUM (Centre hospitalier université de Montréal) and ETS (Ecole de technologie supérieure), and all participants provided written consent before the start of the experiment.

### Materials

**Fig. 1:**
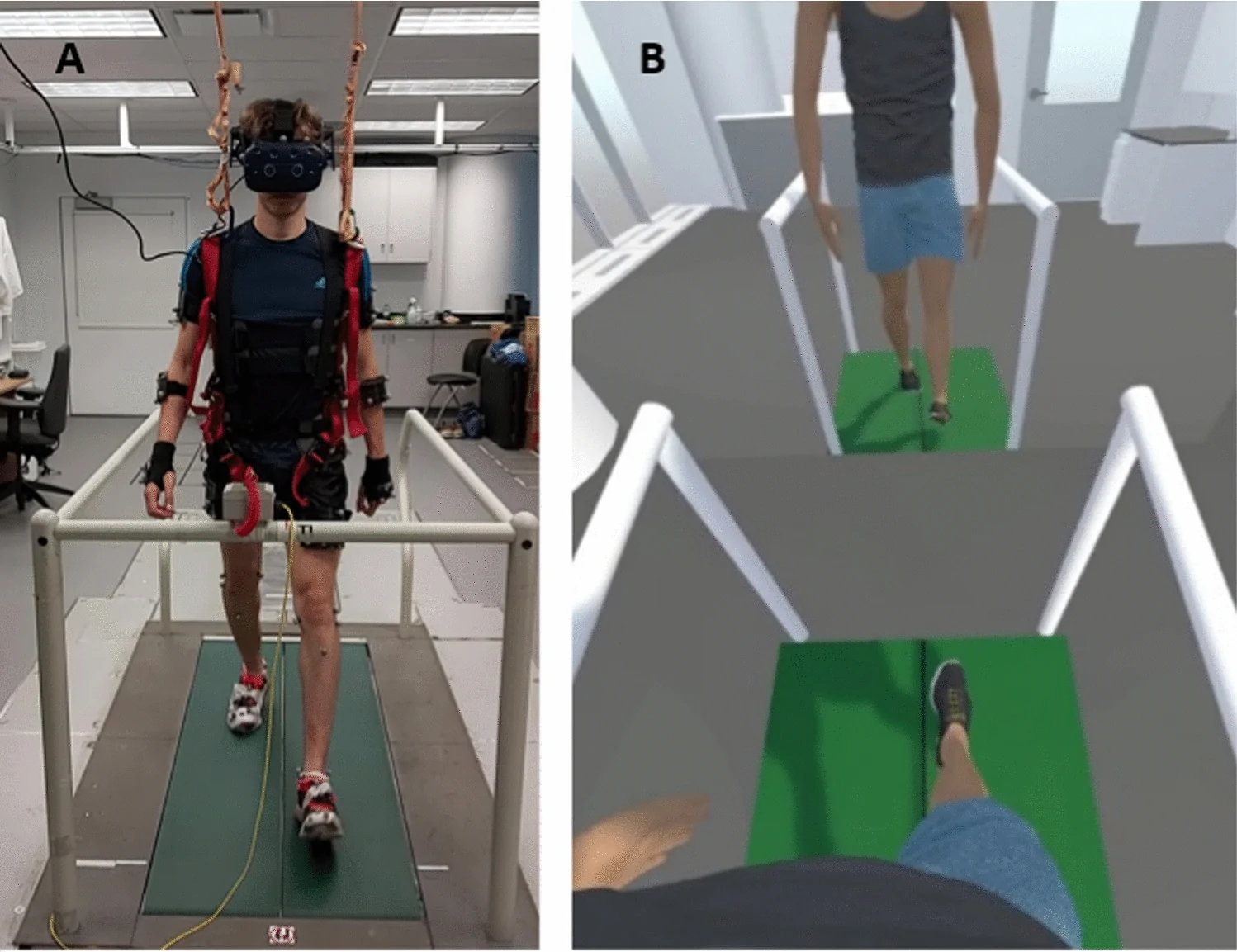
Experimental setup. **A** A participant walking on the treadmill while wearing the motion capture clusters, the Vive Pro Eye, and the safety harness attached to the ceiling. **B** The participant’s avatar walking in the VE in front of a virtual mirror, viewed from a first-person perspective

Participants were asked to walk on a split-belt treadmill (Advanced Mechanical Technology Inc, Waterdown, MA, USA) with both treadmill belts tied at all times. A safety harness was used to prevent and support their weight in the event of a fall due to loss of balance while walking on the treadmill for the duration of the entire experiment (Fig. 1). Kinematic data, recorded at a sampling frequency of 200 Hz, were collected using a 12 Vicon T20-S system (Vicon Motion Systems Ltd, Oxford, UK). Real-time data capture and avatar retargeting in the virtual environment were performed in Vicon Tracker 3.6 using rigid-body marker clusters (14 rigid-body clusters in total) attached with Velcro straps to track the toes, feet, upper legs, pelvis (L5–S1), hands, upper arms, forearms, upper trunk (between the scapulae). In addition, for the post hoc kinematic analysis of joint angles, 8 single reflective markers were placed bilaterally on anatomical landmarks, including the greater trochanter (GT), lateral femoral epicondyle (LEP), lateral malleolus (LM), and the midpoint of the heel. All markers were applied prior to the start of the experiment and remained in place throughout the session. Vicon Pegasus Advanced 1.2.2 then retargeted limb positions and orientations onto a virtual avatar displayed in the virtual environment (VE) in real-time. The avatar, created with MakeHuman 1.20 (MakeHuman), was scaled based on participants’ body height and weight and gender-matched. Both male and female avatars were outfitted in blue shorts, a grey T-shirt, and blue trainers (Fig.  1) Visualized through an HTC VIVE Pro Eye head-mounted display (HMD) with a first-person perspective (1PP) relative to the avatar, the VE featured a replica of the research lab. The lab included a virtual mirror positioned 1.0m in front of the virtual treadmill. Unity 3D (Unity) game engine version 2019.3.13f1 was used to create the VE, ensuring that the virtual treadmill and safety railings matched the dimensions of the real-world environment for a safe walking experience in the VE.

### Gait symmetry distortion model

**Fig. 2:**
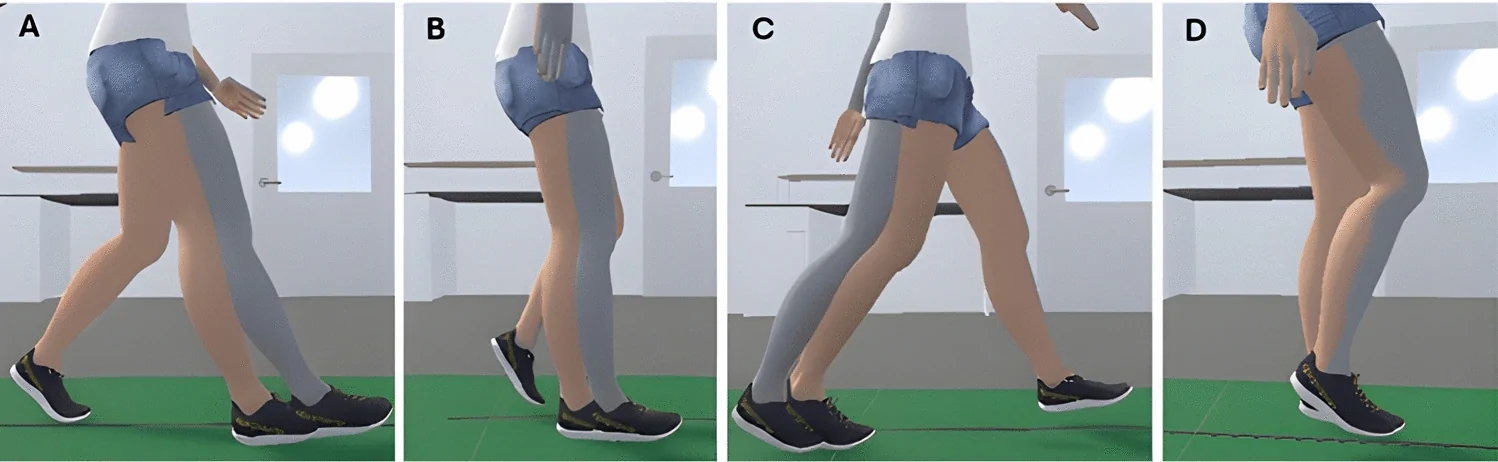
Right step length distortion. In this illustration, the grey avatar shows the participant’s actual leg position, while the opaque avatar depicts the altered position applied to the right lower limb. Participants viewed their avatars from a first-person perspective (1PP) and could not see the translucent avatar. **A** The avatar’s right limb is in mid-swing. At this point, the AP offset of the ankle is zero and begins to increase linearly along the AP axis of the tibia. **B** At the moment of right heel-strike, the ankle offset reaches its maximum positive value and then starts to decrease. **C** The avatar’s right heel lifts off the ground (heel off). The ankle offset reaches its maximum negative value and then decreases, returning to zero offset at mid-swing (**A**). **D** As the right limb progresses into the next swing phase, the offset decreases and returns to zero at mid-swing, completing the cycle

To introduce asymmetry in the avatar’s gait in real time, we implemented an algorithm that progressively modifies the step length of one leg during walking. This manipulation was achieved by applying a time-varying spatial offset between the virtual ankle and the user’s actual ankle position along the anteroposterior (AP) axis of the tibia (see Fig.  2). The magnitude of the offset was defined as a percentage of the participant’s average step length, calculated from a 2 min baseline walking trial.

The offset profile followed a gait-phase-dependent pattern. It increased steadily starting from mid-swing, identified as the point where the ankle aligns vertically with the hip during forward leg motion, causing the avatar’s ankle to move forward along the AP axis until the moment of heel strike (Fig.  2A). At heel strike, the offset reversed direction and began decreasing (Fig.  2B), becoming negative by midstance. This negative offset then increased in magnitude until heel-off (Fig.  2C), after which it gradually returned to zero by the next mid-swing. The ratio between forward and backward displacements was calibrated individually, based on each participant’s maximum anterior and posterior ankle positions relative to the hip.

The resulting movement of the avatar’s lower limb was generated using inverse kinematics via the FinalIK plugin (RootMotion). To support the sense of embodiment, the onset of the avatar’s (distorted) heel strike remained temporally aligned with the participant’s actual heel strike, thereby preserving visuotactile synchrony (Kokkinara et al. 2015).

### Experimental protocol

Participants were invited to perform the experiment in a single session, which lasted 3 hours and 30 min.

#### Calibration and baseline

Participants first completed a brief treadmill familiarization lasting approximately 5 min, performed without the HMD. This phase ensured that each participant was at ease with walking on the treadmill, provided an opportunity to verify that the reflective marker clusters remained secure, and allowed the determination of a comfortable walking pace. The belt speed began at 1 m/s and was adjusted in increments of 0.1 m/s until participants confirmed that it matched their preferred pace.

Once this preliminary stage was complete, the HMD was fitted and the avatar’s position aligned to the participant’s body using the Vicon Pegasus Advanced system. During calibration, participants were asked to stand upright with feet pointing forward, arms relaxed at their sides, and their gaze directed straight ahead. The virtual body was kept hidden during this process to prevent exposing participants to potential visual discrepancies.

After calibration, participants engaged in a 1- min movement phase designed to induce a sense of embodiment. They alternated between looking down at their limbs and observing themselves in the virtual mirror while performing upper and lower limb movements. Prior work has shown that such active visuomotor engagement can enhance the feeling of ownership over a virtual body (Kokkinara and Slater 2014). Subsequently, participants completed a 5- min treadmill walk while wearing the HMD to familiarize themselves with ambulation in the virtual environment (VE). Participants were instructed to alternate their gaze between their lower limbs and their reflection in the virtual mirror throughout the baseline and experimental phases, to ensure that the avatar’s gait remained visible. This instruction was repeated before each condition. Gaze behavior was not recorded; therefore, adherence to these viewing instructions was not formally quantified.

They were asked to refrain from using the treadmill’s handrails and to notify the experimenters immediately in case of discomfort or symptoms such as dizziness or nausea. The final 2 min of this baseline walk were used to record step length, calculated as the distance in the anteroposterior direction between the heels of the leading and trailing foot at heel contact.

#### Experimental phase

The effects of step length manipulation on gait symmetry were assessed over two different conditions: Dominant-Leg Distortion and Non-Dominant-Leg Distortion. All participants completed both conditions (within-subject design). Each condition comprised three trials and lasted approximately 15 min. The order of the two conditions was counterbalanced across participants to minimize potential order effects.

In both conditions, the avatar’s step length on the manipulated side was progressively altered in 1% increments every 10 strides, reaching a maximum distortion of 30%. Each distortion level lasted for about 10 seconds. The experimental protocol included both ascending trials (0% to 30%) and descending trials (30% to 0%). Only the ascending trials are analyzed in the present study, as they were specifically designed to assess motor adaptation to gradually increasing step length distortion. The descending trials, focused on perceptual thresholds and embodiment, were reported separately (Willaert et al. 2024). Both analyses stem from the same protocol, which was originally designed to address both perceptual and motor aspects of gait distortion under embodiment.

Each condition consisted of three trials, with each trial lasting 5 min. A verbal indication was provided to indicate the end of one trial and the start of the next trial. The instructions given to the participants were to look at the avatar’s head in the mirror after the completion of all levels of distortion. Following this, the avatar’s step length returned to 0%, and the following trial started without stopping the treadmill between trials. The distortion range (0% to 30%) was selected based on insights from four pilot studies. A 30% increase was a substantial distortion at which the avatar’s gait manipulation became clearly noticeable to participants without inducing an extremely unstable gait that may induce discomfort or instability. A 10- min break was provided between each condition. Additionally, a 1- min washout period was implemented at the beginning of each condition to minimize any potential gait adaptations following exposure to step length distortions (Kim 2012; Lauzière et al. 2014). Throughout the washout period, the step length of the avatar remained unaltered, and participants were instructed to observe their avatar by looking down at their body and in the mirror while walking.

### Data analysis

Data analysis for step length, gait symmetry ratio and joint angles was conducted with a custom-written script in MATLAB R2023b (MathWorks, Natick, MA). All statistical analysis was performed in R Studio (version 2023, RStudio, PBC, Boston, MA). The statistical significance level was set at 0.05 for all tests.

#### Gait symmetry

The step length was calculated separately for the dominant and non-dominant legs for each participant across the three trials in both conditions. An average step length was determined for each distortion level (10 steps per distortion level) for both the dominant and non-dominant legs. Subsequently, these average step lengths per distortion level were aggregated to compute an overall mean step length, reflecting data from all participants. To account for individual variations, the step length was normalized by dividing it by the respective leg length of each participant. To further investigate the effect of distortion on gait, a gait symmetry ratio was obtained by dividing the step length of the dominant over the non-dominant leg. A ratio of 1 indicated perfect symmetry.

A Linear Mixed Model (LMM) was used to evaluate the impact of distortion levels on step length and gait symmetry ratio. Separate models were fitted for the step length of the dominant leg and the step length of the non-dominant leg, as well as for the gait symmetry ratio. Each model included two predictors as fixed effects to analyze both main effects and interactions: the Manipulated leg (the avatar’s leg to which the distortion was applied) and the Distortion level. Distortion magnitude was modeled continuously in the LMM. Participants were included as a random intercept to account for repeated measurements within individuals. For descriptive plots and post-hoc summaries, distortion values were additionally grouped into 5% intervals (0%, 5%, 10%, 15%, 20%, 25%, and 30%) to improve readability and limit the number of pairwise comparisons.

Following the LMM analysis, the Spearman correlation coefficients were calculated separately for each condition and for each step length (dominant and non-dominant) to explore the relationship between distortion levels and step length within both conditions. Model assumptions were evaluated using residual diagnostics (Q–Q plots and residuals-versus-fitted plots); residuals were approximately normally distributed with minor deviations in the tails.

#### Joint angles

Joint-angle analyses were included as an exploratory outcome to identify potential compensatory strategies (hip/knee/ankle contributions) that might occur even when step-length symmetry remains unchanged. The hip joint flexion angles were calculated by determining the angle between the thigh segment (consisting of GT and the LEP marker) and the pelvis rigid body. The angle between the thigh and shank segments determined the knee joint angle. The shank segment was defined using markers on the LEP and LM markers. The ankle joint angle was calculated by the angle between the shank and the foot segments, with the foot segment defined by the LM marker and toe rigid bodies (placed in the middle of the phalanges). All joint angles were calculated in the sagittal plane. Lower limb joint angles for the hip, knee, and ankle were calculated for each gait cycle. Similar to the gait symmetry analysis, an initial average was calculated for each participant for each trial and condition across different distortion levels. Subsequently, these individual averages were combined to compute an overall mean across all participants in order to evaluate the impact of the avatar’s step length distortion level on joint angles per condition. The peak knee flexion, peak hip flexion, and peak ankle flexion were analyzed to compare joint angles across conditions. Six separate LMMs were employed, one for each of the three lower body joints for both the dominant and non-dominant legs. Each model included the predictor’s manipulated leg and distortion levels (0%, 5%, 10%, 15%, 20%, 25%, and 30%). To compare joint angles across conditions, the peak knee flexion (during the swing phase), peak hip flexion (during the swing phase), and peak ankle flexion (during the stance phase) were analyzed.

#### Embodiment

Embodiment scores were collected at each distortion level (0–30%, in 3% increments), where participants verbally rated their sense of embodiment using the statement “I feel that the virtual body is my own body” on a 0–7 Likert scale.

In parallel, a detection threshold was established, defined as the distortion magnitude below which participants did not perceive a mismatch between the avatar’s gait and their own gait. This threshold was obtained by asking participant to verbally indicate when they perceived that the avatar’s gait no longer matched their own. The detailed procedure for collecting the embodiment and detection responses is described in Willaert et al. (2024) In the present study, we examined whether embodiment was associated with gait symmetry adaptation to the distortion.An LMM was used to assess whether embodiment ratings predicted gait symmetry ratios, with embodiment score as a fixed effect and a random intercept for each participant to account for repeated measures.

## Results

### Gait symmetry

**Fig. 3:**
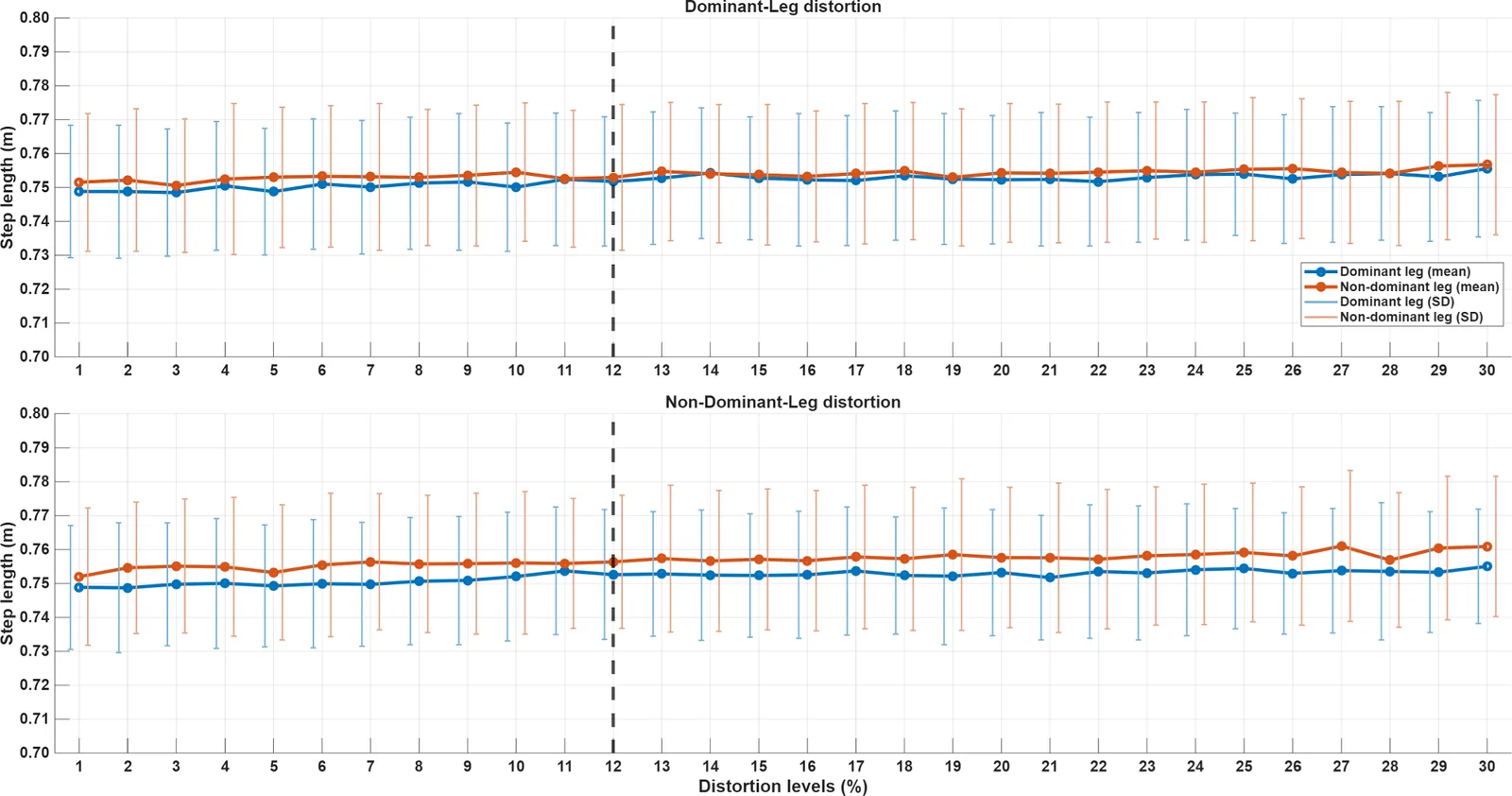
Mean dominant and non-dominant step length results across all 30 distortion levels for both conditions, averaged over all participants. Error bars indicate 1 standard deviation; to reduce overlap, SD is plotted on alternating distortion levels (even levels for dominant-leg series, odd levels for non-dominant-leg series). The vertical dashed line indicates the group-level perceptual threshold, defined as the distortion magnitude at which participants reported detecting the distortion

Figure 3 illustrates the overall mean step length across all participants for each distortion level in both conditions. The results of the LMMs analyzed the effects of the Manipulated limb (the limb where the distortion was applied, either the dominant or non-dominant leg of the participant) on the step length of both the dominant and non-dominant legs. For the step length of the participant’s dominant leg, there were no significant main effects of the Manipulated leg on the dominant step length (SE=0.002,t(776)=0.15,p=0.87). However, the predictor Distortion level demonstrated a significant main effect (SE=0.00007,t(776)=2.98,p=0.002). However, the estimated magnitude of this effect was negligible (marginal R² = 0.003), corresponding to an approximate change of  0.6 cm from 0% to 30% distortion. Additionally, no interaction effects between the two predictors were found. Furthermore, the Spearman correlation analysis indicated no significant relationship between the dominant step length and the distortion levels in the Dominant-leg distortion condition (*ρ = 0.056*, p = 0.10 ) or the Non-Dominant-leg distortion condition (*ρ = 0.052*, p = 0.12) , consistent with the very small effect magnitude estimated by the LMM.

**Fig. 4:**
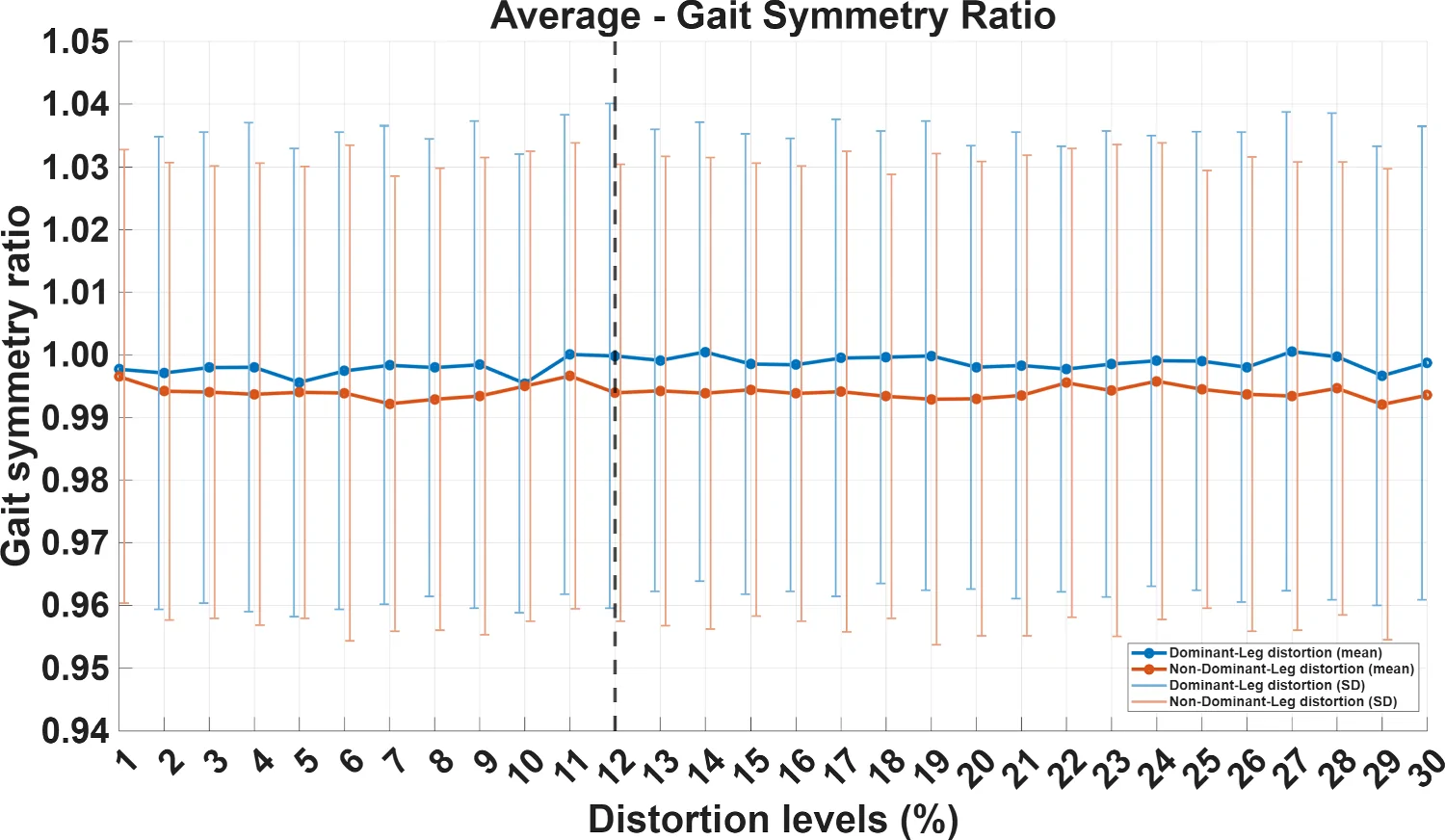
Mean gait symmetry ratio results across all 30 distortion levels for both conditions, averaged over all participants. Error bars denote standard deviation and are plotted at alternating distortion levels to minimize overlap, with even levels shown for the dominant-leg series and odd levels for the non-dominant-leg series. The vertical dashed line indicates the group-level perceptual threshold, defined as the distortion magnitude at which participants first reported detecting the distortion

For the step length of the participant’s non-dominant leg, the LMM results showed no significant main effects for Manipulated leg (SE=0.002, t(374)= 0.09, p = 0.93). However, the Distortion level (SE=0.0001, t(374)= 2.09, p = 0.03) did demonstrate a significant main effect. As with the dominant leg, the estimated magnitude of this effect was negligible (marginal R² = 0.0045), corresponding to an approximate change of  0.6 cm from 0% to 30% distortion. No interaction effects between both predictors were detected. The Spearman correlation analysis revealed no significant relationship between the Dominant-leg distortion condition (*ρ = 0.038*, p = 0.28 ) or the Non-Dominant-leg distortion condition (*ρ = 0.053*, p = 0.12). Figure 4 illustrates the gait symmetry ratio across all the 30% distortion levels for both conditions. The gait symmetry ratio LMM revealed no significant main effects for either the Manipulated leg (SE=0.002, t(374)= -0.35, p=0.72) or the Distortion level (SE=0.0008, t(374)= 0.5, p= 0.55). No significant interaction effects were observed for the gait symmetry ratio. These results indicate that the incrementally introduced step length distortion levels did not meaningfully influence step length (despite statistically detectable but negligible effects in the LMMs) and the gait symmetry ratio across either condition.

As a secondary check, we examined the descending phase (30%*→ *0%) and observed the same overall pattern (no evidence of a self-avatar follower effect), despite comparable embodiment during this phase reported in Willaert et al. (2024); we therefore do not report a full parallel analysis; full statistical results are provided in the nn Materials.

### Joint angles

**Fig. 5:**
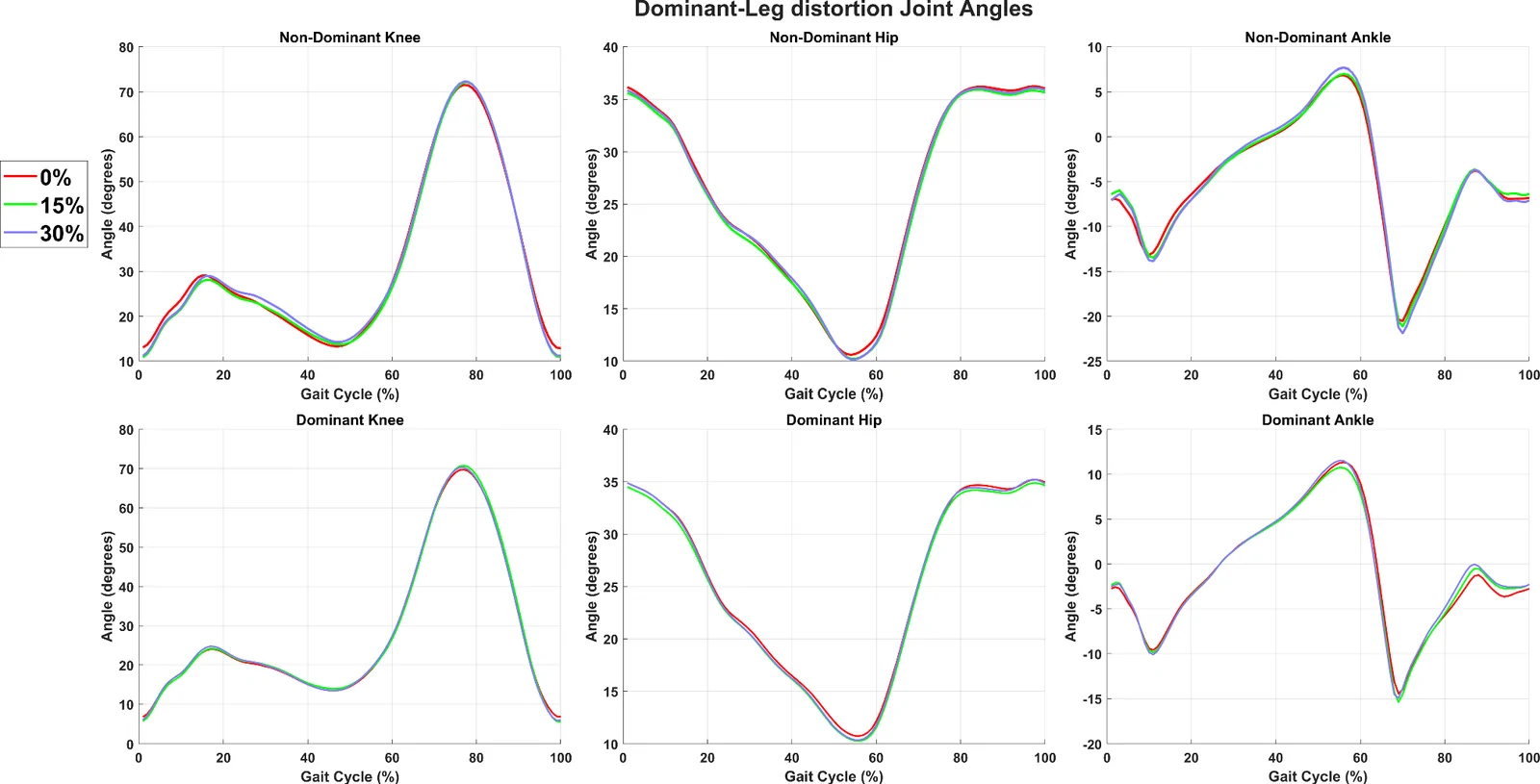
Joint peak angles for the knee, hip, and ankle joints across the gait cycles for the condition Dominant-Leg distortion. The results shown are for a single participant and reflect the mean trends. The distortion levels at 0%, 15%, and 30% are represented by different colours

**Fig. 6:**
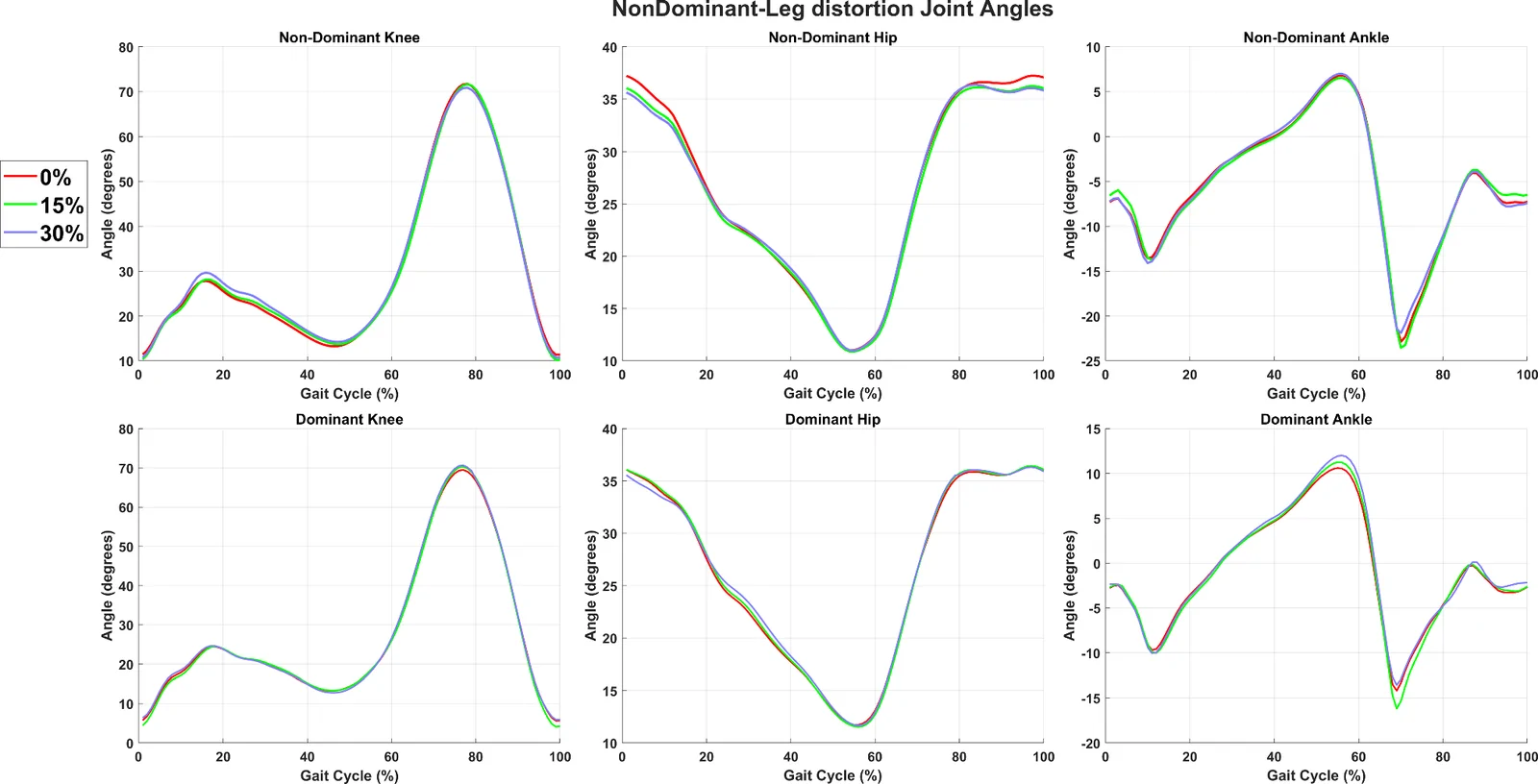
Joint peak angles for the knee, hip, and ankle joints across the gait cycles for the condition NonDominant-Leg distortion. The results shown are for a single participant and reflect the mean trends. The distortion levels (0%, 15%, 30%) are represented by different colours

For the joint angles analysis, the dataset included data from 20 participants. Data from an additional 9 participants were excluded entirely from the joint-angle analysis due to persistent missing marker trajectories during data capture across multiple trials, which prevented reliable reconstruction of lower-limb kinematics and joint angles. While this resulted in a reduced sample size (n=20) for the joint angle analysis, the remaining dataset preserved the necessary structure, sufficient valid trials and consistency across conditions to support a robust repeated measures approach. Figures 5 and 6 illustrate the average joint angles across the gait cycle for the hip, knee, and ankle joints of both the dominant and non-dominant limbs, with Fig.  2 illustrating the Dominant-Leg distortion condition and Fig. 5 depicting the NonDominant-leg distortion condition. Different distortion levels are represented by various colours for both conditions.

The results of the linear mixed model (LMM) analysis revealed no significant main effects for the predictors Manipulated leg or Distortion level on peak knee flexion for either the dominant or non-dominant leg, and no interaction effects were observed. Similarly, no significant main or interaction effects were identified for Manipulated leg or Distortion level on peak ankle flexion in both the dominant and non-dominant legs.

For dominant hip peak flexion, while distortion levels (SE = 0.01, t(533) = 0.47, p = 0.63) did not yield a significant main effect, a significant main effect was observed for the predictor Manipulated leg (SE = 0.49, t(257) = 2.78, p = 0.005). However, the effect size, as indicated by the marginal R² (R² = 0.0061), was very small, suggesting that while statistically significant, the effect has minimal practical relevance within the context of the avatar’s step length distortion.

### Embodiment

The results of the LMM analysis revealed no significant association between embodiment ratings and gait symmetry (SE = 0.0001, t(608) = 0.38, p = 0.70). This suggests that variations in the sense of embodiment did not modulate gait behavior when being exposed to step length distortion experienced through an embodied avatar.

## Discussion

### Gait symmetry

The first objective of this study was to evaluate the influence of step length distortion, as presented by an embodied avatar, on the step length of healthy individuals during treadmill walking. Contrary to our initial hypothesis (H1), our findings revealed no changes in gait symmetry among healthy participants during treadmill walking. These findings offer an important extension to prior work by Burin et al. (2019) who demonstrated that users’ motor output could be modulated during continuous, rhythmic movements of the upper-limb. While we did not observe a comparable follower effect in the context of gait, our results highlight how the underlying control mechanisms of gait may limit the extent to which the follower effect generalizes. In this sense, our study complements the work from Burin et al. (2019) by revealing a potential boundary condition for the follower effect: while rhythmic movement may be sufficient for spontaneous adaptation in upper-limb tasks, the automaticity and proprioceptive dominance of gait may limit the extent of similar modulation through avatar-based distortions, under the present conditions.

Gait is a highly automatic motor task controlled by the central nervous system and central pattern generators, which produce rhythmic coordinated movements without necessitating conscious effort(Malone and Bastian 2010; Clark 2015). As described during the studies involving reaching tasks, the discrepancy between the visual information presented (the avatar’s altered step length) and the proprioceptive feedback being received from the participant’s own body movements potentially created a sensory conflict. However, the findings of the current study suggest that in the context of gait, proprioceptive feedback about the body’s real movement and position may have reduced the influence of the distorted avatar, resulting in stable gait patterns. In contrast, upper limb movements are often visually guided and rely on continuous sensory feedback and cortical control for fine motor skills and adjustments (Miall and Wolpert 1996). Visual feedback plays a critical role in guiding precise hand and arm actions during voluntary upper body movements, often carrying greater perceptual weighting than proprioceptive input (Brenner et al. 2023). Thus, under the present conditions, the sensory conflict created by the spatial distortion of the avatar may be more influential in visually dominant tasks like reaching than in proprioceptively dominant tasks like gait.

In the current study, the protocol was designed to gradually alter the step length of the avatar over time without the participants’ explicit knowledge. Participants were intentionally not instructed to synchronize with or mirror the avatar’s step length, allowing us to observe whether gait adjustments would occur spontaneously during treadmill walking. Other studies have also examined the effect of implicitly modified visual feedback in healthy participants, though not through the use of an avatar. These studies reported significant differences in gait symmetry when visual feedback was manipulated using bar graphs that represent, in real time, the step length of each foot(Kim 2012; Tobar et al. 2018). This distortion created a perceived asymmetry, prompting participants to adjust their walking patterns away from symmetry to align with the visual feedback, even without explicit instructions. Although implicit feedback in these studies, delivered through direct visual indicators such as bar graphs, has been shown to elicit changes in gait symmetry, we did not find evidence of meaningful gait adaptation in response to the avatar’s distorted step length. One possibility is that so-called implicit visual feedback paradigms (e.g., step-length bar graphs) still provide an explicit representation of the relevant error (left–right step-length asymmetry) with a clear reference, making it straightforward to interpret and translate into a concrete gait adjustment. In contrast, our avatar-based manipulation did not provide an external reference for symmetry; only the avatar’s step length was altered. Without an external reference for symmetry, participants could only rely on the visual appearance of their own lower limbs (first-person view and mirror) relative to their proprioceptive sense of stepping. In this context, small unilateral exaggerations may not yield a stable or easily interpretable error signal: the visual difference is subtle, step-to-step variability is natural, and there is no explicit “zero-asymmetry” reference against which to judge error. Consistent with this, our prior threshold work showed that distortions can remain below conscious detection up to  12% (Willaert et al. 2024). As the distortion increased beyond this range, participants became able to consciously detect the manipulation, indicating a visuo–proprioceptive mismatch large enough to be noticed and potentially serve as an error signal (Kim 2012; Kim and Mugisha 2014; Mazzoni and Krakauer 2006; Taylor et al. 2014). However, for adaptation to occur, the discrepancy must also be interpreted as self-relevant and actionable. In our setup, participants could walk successfully while maintaining overall visuomotor coherence, so the distortion may have been attributed to the avatar rather than treated as feedback about one’s own gait, leading to downweighting of the distorted visual cue. As such, we did not observe a meaningful adaptation toward the avatar’s step length. Future work could increase actionability by adding an external reference (e.g., step-length targets or explicit error displays) or by manipulating visual salience of foot placement; however, such approaches may engage more goal-directed tracking than the spontaneous follower effect targeted here.

The second objective of this study was to assess whether the detection threshold influences the changes in gait of the participants in response to the movements of the avatar. The results indicated that the avatar’s distortion had no meaningful effect on gait symmetry, and consequently, the detection threshold did not influence gait changes. This lack of changes in gait symmetry supports the rejection of the second hypothesis (H2). Furthermore, findings from our previously reported work demonstrated that the sense of embodiment was negatively correlated with the level of distortion: as the distortion increased, embodiment decreased (Willaert et al. 2024). This suggests that while participants’ sense of embodiment was sensitive to the level of step length distortion, gait symmetry showed minimal change.

Recent VR studies have similarly reported dissociations between subjective embodiment and other outcomes. Embodiment strength did not necessarily scale with perceptual effects such as kinaesthetic illusions (Dupraz et al., 2024) or with neural responses during motor imagery (Vagaja et al. 2024). In studies involving altered avatar morphology, ownership, agency, and motor/postural outcomes were also not always directly coupled (Raoul et al. 2024; Vallageas et al. 2024). Similarly, Willaert et al. (Willaert et al. 2026) showed that a strong sense of embodiment toward a spatially distorted avatar did not lead participants to align their movements with the avatar; instead, they actively counteracted the distortion during reaching tasks performed without visual target. In line with this work, our results show that although participants maintained a sense of embodiment toward the avatar, higher embodiment ratings were not associate with greater gait adaptation to the step-length distortion.

Lastly, this study involved healthy participants whose gait is optimized for balance and efficiency and may be less readily modulated through visual distortions. In contrast, stroke patients, who often exhibit asymmetrical gait patterns and, depending on the lesion location, proprioceptive deficits, tend to rely more on visual feedback (Bernard-Espina et al. 2021; Bonan et al. 2004). As a result, stroke patients might respond differently to interventions involving distorted embodied avatar gait movements. Moreover, previous research suggests that stroke individuals often exhibit a stronger sense of ownership over the affected limb, likely because disruptions in sensory feedback and motor commands reduce their connection to the limb (Burin et al. 2015; Jenkinson et al. 2020). Despite this, similar levels of proprioceptive drift and comparable, though less vivid, embodiment have been observed in stroke patients and healthy individuals in immersive VR settings (Llorens et al. 2016; Borrego et al. 2019). Future studies should investigate how stroke patients respond to avatar-based distorted feedback during gait, designed to address symmetrical gait, and how this affects their sense of embodiment. Exploring the relationship between altered visual feedback provided by self-avatars, the influence on gait symmetry and the sense of embodiment in stroke patients could provide valuable insights for refining and optimizing avatar-based rehabilitation protocols tailored to this population.

### Limitations

In this protocol, participants took part in a detection threshold assessment, which required them to identify whether their avatar’s gait accurately reflected their own (results published in Willaert et al. (Willaert et al. 2024). This process may have influenced participants’ adaptation to the avatar’s movements, as they were made aware of potential distortions in the avatar’s gait. Although their attention was directed toward observing differences in the avatar’s gait, they were not explicitly informed about the specific distortion in step length. Moreover, healthy individuals do not typically observe their lower body while walking. In our VE, participants could see their entire body, which may have increased their awareness of their movements and potentially disrupted their natural gait. However, in a rehabilitation setting, similar attention to gait distortions would be necessary to ensure that patients observe the avatar’s gait distortions.

Another limitation is that participants walked on a treadmill with fixed speeds, which may have constrained gait by enforcing a consistent pace and reducing natural variability like stopping or pausing. While treadmill walking is widely accepted to represent natural gait in controlled experiments (Lee and Hidler 2008; Dingwell et al. 2000), these constraints might have limited gait adaptation to the step length distortion. In particular, at a constant belt speed, increasing step length may require compensatory changes in cadence, which could have constrained the expression of a follower effect. Therefore, it remains unclear whether the same responses would be observed during overground walking, where speed and stride parameters can vary more freely.Nevertheless, previous studies have shown that visuomotor adaptation occurs effectively during treadmill walking, with participants on adjusting step length symmetry in response to real-time visual feedback (Sato et al. 2022; Kim 2012; Tobar et al. 2018). However, because the present study did not demonstrate an adaptation to the embodied-avatar step-length manipulation, our results do not validate this specific treadmill and self-avatar setup as a platform to study visuomotor adaptation. Further work is needed to determine under which conditions and populations this paradigm reliably elicits adaptation.

Furthermore, we applied the step-length distortion to one limb at a time to introduce a controlled left–right asymmetry, but we did not test distortions applied simultaneously to both legs. Because we did not observe the expected adaptation under unilateral manipulation, we cannot determine whether other distortion structures (e.g., bilateral or more whole-gait manipulations) might be more effective at eliciting a self-avatar follower effect. Future work should examine how bilateral distortion influences adaptation.

Finally, we note that null-hypothesis significance testing alone cannot prove the absence of an effect. While our results do not support a robust self-avatar follower effect under the present conditions, it remains possible that the avatar-based manipulation did not provide a sufficiently salient/actionable error signal to elicit measurable adaptation.

### Future work

Our recent findings in gait show that the SoE does not significantly break after detecting distortions introduced gradually, indicating that participants still perceive the avatar as part of their body despite the misalignment (Willaert et al. 2024). However, in the current study, without observed gait adjustments, it is unclear how this maintained embodiment influences motor behaviour post-detection. In upper body tasks, where motor adjustments have been observed in response to distortions (Burin et al. 2019; Gonzalez-Franco et al. 2020), exploring the influence of detection thresholds could provide valuable insights into the relationship between sensory awareness, motor adaptation and the SoE. Future research should explore this relationship in tasks where motor adjustments are more evident, such as upper body movements, to determine whether participants actively correct perceived errors (Kusafuka et al. 2022) or gradually adapt their movements to align with the distortion (Abeele and Bock 2001).

Moreover, it remains unclear how motor adaptation persists in tasks involving lower limb movements when the avatar is spatially misaligned. Ongoing work is investigating how to better understand these dynamics, the effects of the spatial distortion are being examined in lower limb movements under conditions that challenge stability, such as balancing on one leg, to isolate motor responses to avatar distortions. Comparing these effects between the limbs could shed light on differences in the strength of the movement adaptations and the role of the sense of embodiment in these distinct motor tasks.

In the current study, we applied a unilateral step length distortion, creating a visual asymmetry in only one limb. Future studies could investigate whether bilateral distortions that introduce asymmetries across both legs are more effective in eliciting gait adaptation than unilateral distortions. In the unilateral distortion, the non-distorted leg provided a consistent visual reference, which may have led participants to maintain their natural symmetrical gait rather than adjust to the manipulated feedback. This preserved congruence on one side could have reduced the salience of the overall asymmetry, particularly in healthy individuals who had no underlying motor impairment that would necessitate gait correction. In contrast, applying bilateral distortions that introduce asymmetries across both legs may remove this stable reference. Without a congruent side to rely on, the visuomotor conflict may become more pronounced. Testing such configurations would help clarify whether bilateral distortions are more effective at influencing gait behavior in immersive virtual environments.

## Conclusion

This study suggests that healthy gait patterns show limited adaptation to visual distortions introduced by an embodied avatar in a virtual environment. Despite the fact that participants perceived the step length distortions, their gait symmetry showed no meaningful change, and they did not adapt their movements to align with the avatar’s distorted step length. The absence of spontaneous adjustments suggests that the sensory conflict generated by the avatar’s distorted step length was not sufficient to consistently override participants’ reliance on intrinsic sensory feedback, thereby limiting spontaneous alignment with the avatar’s movements and leaving the automaticity of healthy gait unchanged. Future research should explore these dynamics in populations with gait impairments, such as stroke patients, who could benefit from tailored rehabilitation protocols incorporating distorted self-avatars.

## Supplementary Information

Below is the link to the electronic supplementary material.

Supplementary file 5 (docx 41 KB)

Supplementary file 5 (docx 41 KB)

Supplementary file 5 (docx 41 KB)

Supplementary file 5 (docx 41 KB)

Supplementary file 5 (docx 41 KB)

## Data Availability

Data may be available from the corresponding author upon request and subject to approval by the relevant research ethics board and applicable institutional regulations.
