# Supplementary material for: Effect of unilaterally manipulating the step length of an embodied avatar on gait symmetry in healthy adults
Source: Virtual Real. 2026 Jun 25;30(4):162. doi: 10.1007/s10055-026-01406-2 (PMC13558336; doi:10.1007/s10055-026-01406-2)
Supplement: Supplementary file 1 [file 10055_2026_1406_MOESM5_ESM.docx]

**LMM Results for gait symmetry ratio Descending trials**

Table 1: Model Summary for Gait Symmetry Ratio

| **Term** | **Value** | **Std. Error** | **DF** | **t-value** | **p-value** |
| --- | --- | --- | --- | --- | --- |
| (Intercept) | 1.0001432 | 0.0036204 | 374 | 276.249000 | 0.0000000 |
| Distortion_Level | -0.0001113 | 0.0000734 | 374 | -1.516182 | 0.1303179 |
| Manipulated Leg | -0.0052219 | 0.0019825 | 374 | -2.634061 | 0.0087874 |
| Distortion_Level × Manipulated Leg | 0.0001639 | 0.0001093 | 374 | 1.500018 | 0.1344533 |

**Effect Sizes**

- Marginal R² (Fixed Effects): 0.0064
- Conditional R² (Fixed + Random Effects): 0.7996

**Post-hoc Results**

Table 2: Post-hoc Comparisons for Gait Symmetry Ratio

| **Contrast** | **Estimate** | **SE** | **df** | **t-ratio** | **p-value** |
| --- | --- | --- | --- | --- | --- |
| Dominant - (N-Dominant) | -0.0001639 | 0.0001093 | 374 | -1.500018 | 0.1345 |

**LMM Results for Step length NonDominant – Descending**

Table 3: Model Summary for NonDominant step length Descending

| **Term** | **Value** | **Std. Error** | **DF** | **t-value** | **p-value** |
| --- | --- | --- | --- | --- | --- |
| (Intercept) | 0.7522370 | 0.0077157 | 374 | 97.4940715 | 0.0000000 |
| Distortion_Level | 0.0001463 | 0.0000740 | 374 | 1.9764045 | 0.0488433 |
| Manipulated Leg | 0.0003358 | 0.0021890 | 374 | 0.1534188 | 0.8781508 |
| Distortion_Level × Manipulated Leg | -0.0000264 | 0.0001185 | 374 | -0.2231268 | 0.8235586 |

**Effect Sizes**

- Marginal R² (Fixed Effects): 0.0010
- Conditional R² (Fixed + Random Effects): 0.9571

**Post-hoc Results**

Table 4: Post-hoc Comparisons for NonDominant step length Descending

| **Contrast** | **Estimate** | **SE** | **df** | **t-ratio** | **p-value** |
| --- | --- | --- | --- | --- | --- |
| Dominant - (N-Dominant) | 2.64e-05 | 0.0001185 | 374 | 0.2231268 | 0.8236 |

**LMM Results for Step length Dominant – Descending**

Table 5: Model Summary for Dominant step length Descending

| **Term** | **Value** | **Std. Error** | **DF** | **t-value** | **p-value** |
| --- | --- | --- | --- | --- | --- |
| (Intercept) | 0.7508594 | 0.0079938 | 374 | 93.9307724 | 0.0000000 |
| Distortion_Level | 0.0000999 | 0.0000685 | 374 | 1.4591634 | 0.1453595 |
| Manipulated Leg | -0.0020571 | 0.0019904 | 374 | -1.0335278 | 0.3020248 |
| Distortion_Level × Manipulated Leg | 0.0000239 | 0.0001083 | 374 | 0.2204974 | 0.8256041 |

**Effect Sizes**

- Marginal R² (Fixed Effects): 0.0010
- Conditional R² (Fixed + Random Effects): 0.9658

**Post-hoc Results**

Table 6: Post-hoc Comparisons for Dominant step length Descending

| **Contrast** | **Estimate** | **SE** | **df** | **t-ratio** | **p-value** |
| --- | --- | --- | --- | --- | --- |
| Dominant - (N-Dominant) | -2.39e-05 | 0.0001083 | 374 | -0.2204974 | 0.8256 |

**Correlation Between Distortion Levels and Step Length – Descending**

Table 7: Pearson Correlation

| **Trial** | **Leg** | **Correlation (NonDominant)** | **p-value (NonDominant)** | **Correlation (Dominant)** | **p-value (Dominant)** |
| --- | --- | --- | --- | --- | --- |
| Descending | Dominant | 0.058 | 0.088 | 0.042 | 0.220 |
| Descending | N-Dominant | 0.034 | 0.313 | 0.043 | 0.208 |

**LMM Results for Hip joint angles Dominant – Descending**

Table. Model Summary for Dominant Joint Angle Hip Descending

| **Term** | **Estimate** | **Std. Error** | **df** | **t-value** | **p-value** |
| --- | --- | --- | --- | --- | --- |
| (Intercept) | 31.7573758 | 1.7270849 | 257 | 18.3878484 | 0.0000000 |
| Distortion_Level | 0.0046316 | 0.0105007 | 257 | 0.4410720 | 0.6595317 |
| Manipulated Leg | 1.0686541 | 0.3332976 | 257 | 3.2063060 | 0.0015148 |
| Distortion_Level × Manipulated Leg | -0.0074605 | 0.0163704 | 257 | -0.4557341 | 0.6489661 |

**Effect Sizes**

- Marginal R² (Fixed Effects): 0.0039
- Conditional R² (Fixed + Random Effects): 0.9675

**Post-hoc Results**

Table: Post-hoc Comparisons for Dominant Joint Angle Hip Descending

| **Contrast** | **Estimate** | **SE** | **df** | **t-ratio** | **p-value** |
| --- | --- | --- | --- | --- | --- |
| Dominant - (N-Dominant) | 0.0074605 | 0.0163704 | 257 | 0.4557341 | 0.6490 |

**LMM Results for Hip joint angles NonDominant -Descending**

Table: Model Summary for NonDominant Joint Angle Hip Descending

| **Term** | **Estimate** | **Std. Error** | **df** | **t-value** | **p-value** |
| --- | --- | --- | --- | --- | --- |
| (Intercept) | 32.5256337 | 1.8877135 | 257 | 17.2301749 | 0.0000000 |
| Distortion_Level | 0.0052720 | 0.0106466 | 257 | 0.4951754 | 0.6208994 |
| Manipulated Leg | 0.5859623 | 0.3361247 | 257 | 1.7432881 | 0.0824792 |
| Distortion_Level × Manipulated Leg | -0.0011246 | 0.0168350 | 257 | -0.0668001 | 0.9467927 |

**Effect Sizes**

- Marginal R² (Fixed Effects): 0.0012
- Conditional R² (Fixed + Random Effects): 0.9766

**Post-hoc Results**

Table: Post-hoc Comparisons for NonDominant Joint Angle Hip Descending

| **Contrast** | **Estimate** | **SE** | **df** | **t-ratio** | **p-value** |
| --- | --- | --- | --- | --- | --- |
| Dominant - (N-Dominant) | 0.0011246 | 0.0168350 | 257 | 0.0668001 | 0.9468 |

**LMM Results for Knee Joint angles NonDominant - Descending**

Table: Model Summary for for NonDominant Joint Angle Knee Descending

| **Term** | **Estimate** | **Std. Error** | **df** | **t-value** | **p-value** |
| --- | --- | --- | --- | --- | --- |
| (Intercept) | 69.3141070 | 1.0910340 | 257 | 63.5306562 | 0.0000000 |
| Distortion_Level | 0.0155631 | 0.0081786 | 257 | 1.9029066 | 0.0581702 |
| Manipulated Leg | 0.0452184 | 0.2413565 | 257 | 0.1873511 | 0.8515332 |
| Distortion_Level × Manipulated Leg | 0.0000307 | 0.0130754 | 257 | 0.0023474 | 0.9981289 |

**Effect Sizes**

- Marginal R² (Fixed Effects): 0.0010
- Conditional R² (Fixed + Random Effects): 0.9738

**Post-hoc Results**

Table: Post-hoc Comparisons for NonDominant Joint Angle Knee Descending

| **Contrast** | **Estimate** | **SE** | **df** | **t-ratio** | **p-value** |
| --- | --- | --- | --- | --- | --- |
| Dominant - (N-Dominant) | -3.07e-05 | 0.0130754 | 257 | -0.0023474 | 0.9981 |

**LMM Results for Knee Joint angles Dominant - Descending**

Table: Model Summary for Dominant Joint Angle Knee Descending

| **Term** | **Estimate** | **Std. Error** | **df** | **t-value** | **p-value** |
| --- | --- | --- | --- | --- | --- |
| (Intercept) | 70.1646273 | 1.1457213 | 257 | 61.2405740 | 0.0000000 |
| Distortion_Level | 0.0182370 | 0.0133902 | 257 | 1.3619686 | 0.1744004 |
| LegN-Dominant | 0.6389515 | 0.4207957 | 257 | 1.5184364 | 0.1301336 |
| Distortion_Level × LegN-Dominant | -0.0063916 | 0.0213595 | 257 | -0.2992384 | 0.7650000 |

**Effect Sizes**

- Marginal R² (Fixed Effects): 0.0037
- Conditional R² (Fixed + Random Effects): 0.9098

**Post-hoc Results**

Table: Post-hoc Comparisons for Dominant Joint Angle Knee Descending

| **Contrast** | **Estimate** | **SE** | **df** | **t-ratio** | **p-value** |
| --- | --- | --- | --- | --- | --- |
| Dominant - (N-Dominant) | 0.0063916 | 0.0213595 | 257 | 0.2992384 | 0.7650 |

**LMM Results for Ankle Joint angles NonDominant - Descending**

Table: Model Summary for NonDominant Joint Angle Ankle Descending

| **Term** | **Estimate** | **Std. Error** | **df** | **t-value** | **p-value** |
| --- | --- | --- | --- | --- | --- |
| (Intercept) | 7.2118856 | 1.1234348 | 257 | 6.4194964 | 0.0000000 |
| Distortion_Level | 0.0059622 | 0.0051293 | 257 | 1.1623786 | 0.2461600 |
| LegN-Dominant | -0.2529773 | 0.1520342 | 257 | -1.6639499 | 0.0973412 |
| Distortion_Level × LegN-Dominant | 0.0032753 | 0.0082222 | 257 | 0.3983447 | 0.6907068 |

**Effect Sizes**

- Marginal R² (Fixed Effects): 0.0006
- Conditional R² (Fixed + Random Effects): 0.9902

**Post-hoc Results**

Table: Post-hoc Comparisons for NonDominant Joint Angle Ankle Descending

| **Contrast** | **Estimate** | **SE** | **df** | **t-ratio** | **p-value** |
| --- | --- | --- | --- | --- | --- |
| Dominant - (N-Dominant) | -0.0032753 | 0.0082222 | 257 | -0.3983447 | 0.6907 |

**LMM Results for Ankle Joint angles Dominant - Descending**

Table: Model Summary for Dominant Joint Angle Ankle Descending

| **Term** | **Estimate** | **Std. Error** | **df** | **t-value** | **p-value** |
| --- | --- | --- | --- | --- | --- |
| (Intercept) | 8.1293205 | 1.1657415 | 257 | 6.9735191 | 0.0000000 |
| Distortion_Level | 0.0010766 | 0.0048913 | 257 | 0.2201080 | 0.8259618 |
| LegN-Dominant | 0.0663794 | 0.1467283 | 257 | 0.4523969 | 0.6513647 |
| Distortion_Level × LegN-Dominant | 0.0019728 | 0.0078899 | 257 | 0.2500465 | 0.8027512 |

**Effect Sizes**

- Marginal R² (Fixed Effects): 0.0001
- Conditional R² (Fixed + Random Effects): 0.9916

**Post-hoc Results**

Table: Post-hoc Comparisons for Dominant Joint Angle Ankle Descending

| **Contrast** | **Estimate** | **SE** | **df** | **t-ratio** | **p-value** |
| --- | --- | --- | --- | --- | --- |
| Dominant - (N-Dominant) | -0.0019728 | 0.0078899 | 257 | -0.2500465 | 0.8028 |

**LMM: Effect of Embodiment on Gait Symmetry - Descending**

Table: Model Summary for Embodiment on Gait symmetry Descending

| **Term** | **Estimate** | **SE** | **df** | **t** | **p** |
| --- | --- | --- | --- | --- | --- |
| (Intercept) | 0.9970 | 0.0036 | 608 | 277.97 | < .001 |
| EmbodimentScores | 0.0000362 | 0.0001772 | 608 | 0.20 | .838 |
